# A novel approach to microsurgical teaching in head and neck surgery leveraging modern 3D technologies

**DOI:** 10.1038/s41598-023-47225-2

**Published:** 2023-11-20

**Authors:** Manuel Weber, Joy Backhaus, Rainer Lutz, Christopher-Philipp Nobis, Samuel Zeichner, Sarah Koenig, Marco Kesting, Manuel Olmos

**Affiliations:** 1 Department of Oral and Cranio-Maxillofacial Surgery, Friedrich-Alexander-Universität Erlangen-Nürnberg, Glückstrasse 11, 91054 Erlangen, Germany; 2 Institute of Medical Teaching and Medical Education Research, University Hospital of Würzburg, Würzburg, Germany; 3 MFR Research Foundation, New York, USA; 4 Friedrich-Alexander-Universität Erlangen-Nürnberg (FAU), Erlangen, Germany

**Keywords:** Oncology, Surgical oncology

## Abstract

The anatomically complex and often spatially restricted conditions of anastomosis in the head and neck region cannot be adequately reproduced by training exercises on current ex vivo or small animal models. With the development of a Realistic Anatomical Condition Experience (RACE) model, complex spatial-anatomical surgical areas and the associated intraoperative complexities could be transferred into a realistic training situation in head and neck surgery. The RACE model is based on a stereolithography file generated by intraoperative use of a three-dimensional surface scanner after neck dissection and before microvascular anastomosis. Modelling of the acquired STL file using three-dimensional processing software led to the model’s final design. As a result, we have successfully created an economical, sustainable and realistic model for microsurgical education and provide a step-by-step workflow that can be used in surgical and general medical education to replicate and establish comparable models. We provide an open source stereolithography file of the head-and-neck RACE model for printing for educational purposes. Once implemented in other fields of surgery and general medicine, RACE models could mark a shift in medical education as a whole, away from traditional teaching principles and towards the use of realistic and individualised simulators.

## Introduction

In recent decades, improvements in technology have led to a significantly wider range of indications for microvascular anastomosed free tissue transfer^1^. In contrast, educational research and the development of new models in microsurgical teaching have progressed rather slowly^2^. Due to the length of their education, surgical residents are usually exposed to microsurgery at a later stage in their careers. However, as age significantly influences the success and efficiency of learning fine motor skills such as placing a microanastomosis, learning microsurgery at earlier stages of training and at a younger age appears to be beneficial^3,4^.

Current microsurgery teaching is based mainly on simple bench top or cadaveric animal models. The most commonly used and tested cadaveric animal models are chicken thighs or wings, rat aortas and porcine models^5–8^. Javid et al.^5^ established a level of recommendation comparing the various microsurgical simulation and training models in terms of their status of validation, associated studies and level of evidence. Cryopreserved rat aorta, followed by chicken wings and chicken thighs, achieved the highest level of recommendation in comparison^5^. An alternative approach, disfavoured in numerous studies, is the use of live rats for microsurgical teaching. Despite realistic perfusion properties and almost identical rheological conditions compared to human in vivo anastomoses, no evidence-based advantage over the use of preserved arteries could be found in recent literature^9^. In particular, the anatomically complex and often spatially restricted situation of anastomosis in the head and neck region cannot be fully reproduced in practice on a synthetic ex vivo or small animal model.

A fundamental principle of surgical teaching is the simulation of complex surgical procedures, which leads to improved preparation. Therefore, the development and implementation of realistic training models is of great importance. To date, there are no publications on models that realistically simulate the challenges of head and neck microsurgery (microanastomosis in confined spaces, complex spatial configuration of the vessel ends, etc.). First approaches for variable spatial orientation of artificial microsurgical vessels were presented by Alhomer et al.^10^. However, the presented device lacked simulation of spatial anatomical conditions, including the possibility of a realistic hand support. Recently, Byvaltsev et al., published a promising approach for simulation of microsurgical techniques in space-constrained neurosurgery. A surgical model was designed using computed tomographic data. A skull model with various openings was then tree-dimensionally (3D) printed to simulate common surgical approaches. The 3D printed part was then placed as a mask on conventional animal models (chicken thighs and rat cadavers)^11^. A paper by Papavasiliou et al. on simulating restricted access in microanastomosis of deep inferior epigastric perforator flap pedicles to the internal mammary vessels in autologous breast reconstruction described a 3D printed chest wall as a complement to the current chicken leg model^12^. Both Byvaltsev et al., and Papavasiliou et al., restricted the simulation of the surgical approach mainly to a limited region of hard tissue. They can be seen as modular additions to the conventional chicken model.

By implementing advanced 3D surface scanning and printing technology, we aim to develop a training environment for head and neck microanastomosis that simulates microsurgical procedures under realistic, spatially constrained conditions. This will allow medical students and residents to be prepared for challenging surgical conditions as early as possible in their training and prior to real patient contact. In addition, the use of animal models will be avoided by using a synthetic model. In short, the requirements for an adequate microsurgical teaching model are: (1) Realistic simulation of complex spatial-anatomical conditions in head and neck surgery. (2) Realistic haptic properties of the model to mimic real tissue properties. (3) Economic and future-oriented production and use of the model.

We present the development of the Realistic Anatomical Condition Experience (RACE) model and conclude with a workflow for application to other surgical and general medical fields.

## Methods

### RACE model development

**Figure 1:**
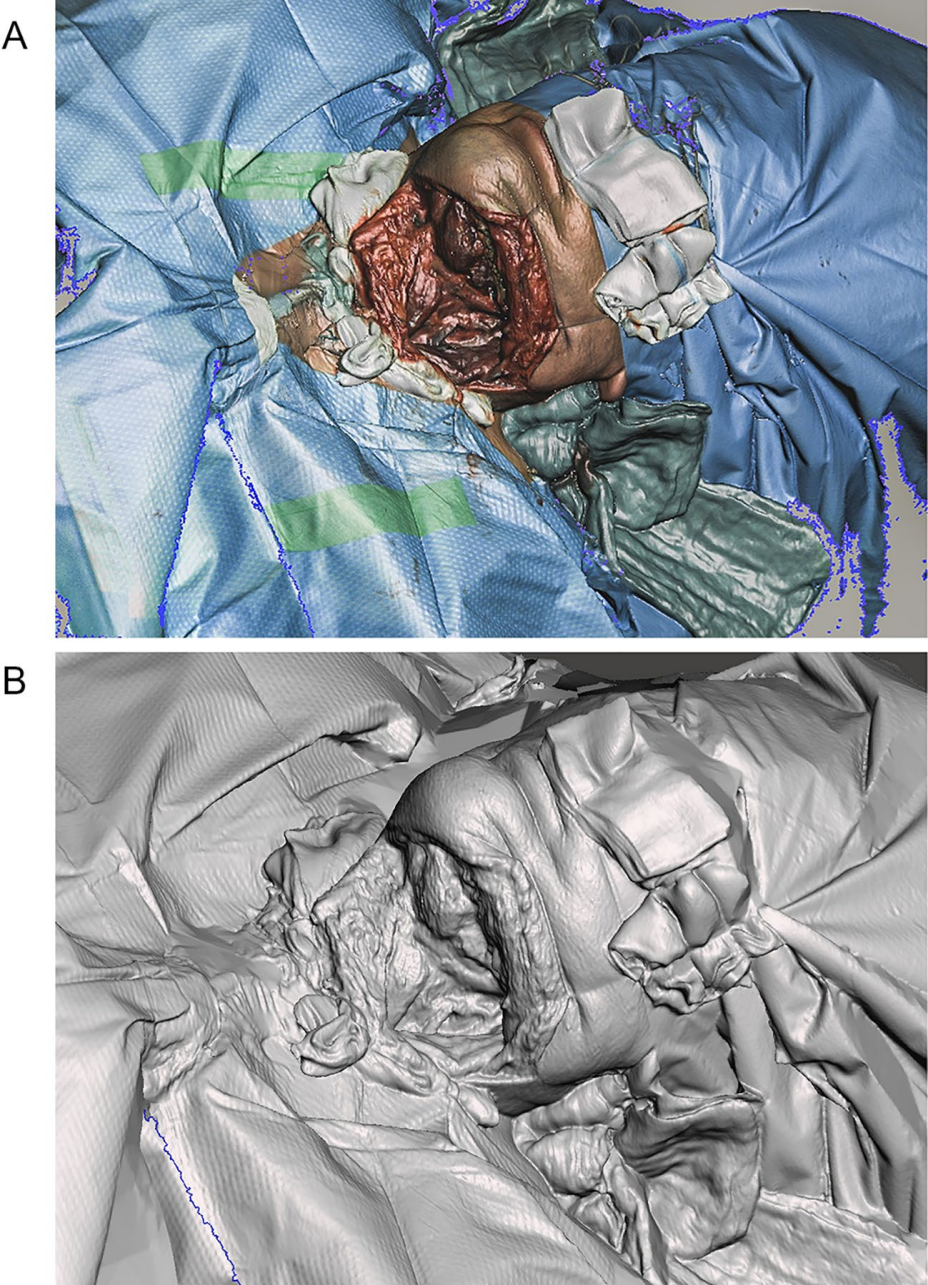
(**A**) Raw data captured by surface scan. Raw data collected after completed neck dissection by intraoperative use of a 3D surface scanner (Co. Artec, Eva, Germany) and exported in STL format. The time required was 10 min. (**B**) Raw data after initial clean-up. Raw data after initial clean-up using open source 3D modelling software (Co. Autodesk, Meshmixer).

**Figure 2:**
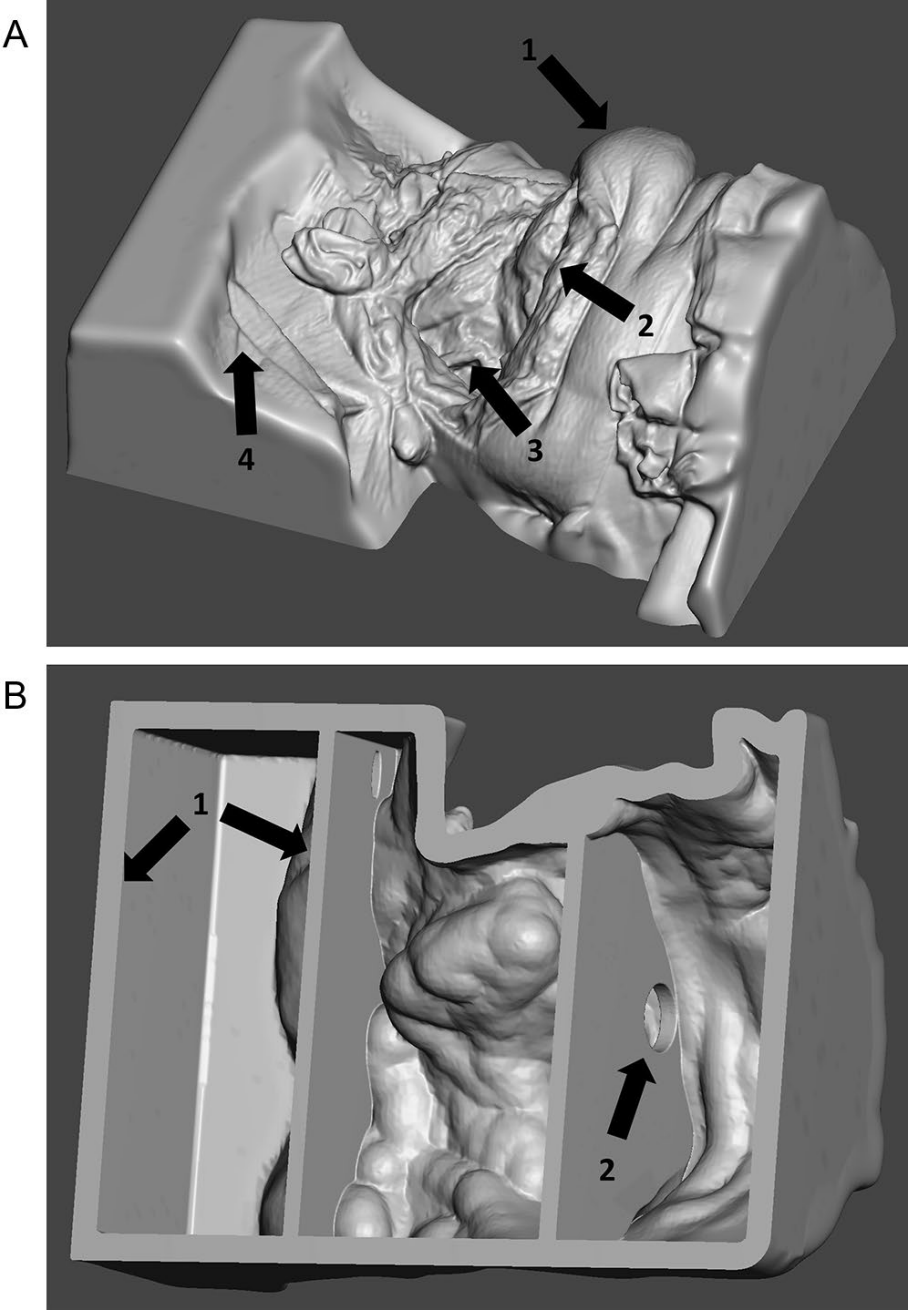
(**A**) STL file of the final RACE model (288 mm*207 mm*115 mm) incl. important anatomical landmarks: (1) patient’s chin, (2) base of the mandible, (3) Superior thyroid artery as possible connecting vessel in head and neck microanastomosis. In this position the fixation devices for the vessels will be inserted, (4) patient’s chest. (**B**) Bottom view of STL file showing the final RACE model. STL file of the final RACE model showing (1) circular supports incl. (2) intermediate partition walls for economic material consumption as well as realistic haptics and flexibility when printed in flexible/elastic material (2) drainage holes for better printability by eliminating suction cups.

Based on the aforementioned problem and model requirements, we considered different options for the 3D acquisition of a surgical site surface after neck dissection. For raw data acquisition we applied a non-contact high-resolution 3D scanner (Co. Artec, Eva, Germany; provided free of charge by: Mr. Frank Forster; Research and Development for Digitalisation and Automation, Siemens AG, Corporate Technology, Germany) after obtaining patient consent (Fig. 1A). Scan time was 10 min, and data were extracted in stereolithography (STL) format. Subsequently, we used open source 3D modelling software for initial clean-up (Fig. 1B) and further modelling (Co. Autodesk, Meshmixer, USA). In terms of the material to be used, we planned the model to be printed in flexible and elastic resin (Co. Formlabs, Flexible Resin 80A and Elastic Resin 50A, USA) in order to realistically replicate human tissue properties and achieve correct haptic feedback. We cropped the scanned surface to the desired and feasible model size at the time of planning and paid special attention to the inclusion of the entire surgical site. As a next step, we negatively extruded the surface (10 mm) to create a volumetric and printable body. Further vertical cuts were made to straighten the inhomogeneous edges. We used the smoothing and inspector tools of the modelling software (Co. Autodesk, Meshmixer, USA) for post-processing and further optimisation. The final RACE model was designed and adapted to a large maximum print volume (288 mm × 207 mm × 115 mm) (Fig. 2A), as prototype development showed that hand support was not sufficiently realistic when smaller models were used. In order to achieve realistic haptics and flexibility, as well as a more economical use of material, we used circular supports including 2 intermediate partitions for the final model design (Fig. 2B, 1). Partitions and side walls were designed with a thickness of 10 mm to match the volumetric body created by extrusion of the surface and provided with drainage holes for better printability (Fig. 2B, 2). We smoothed the edges of the final model again using the robust and shrink smooth modelling tools (Co. Autodesk, Meshmixer, USA).

Once we completed modelling, support structures were calculated and added to allow for a stable printing process using stereolithography. In detail, we set support structure settings to full raft type, 1.0 relative support structure density and 0.7 mm contact point size in the corresponding manufacturer’s software (Co. Formlabs, Preform, USA). Internal support structures were enabled and printing was carried out exploiting maximum print volume. The outcome was satisfactory and replicable. We carried out stereolithography using two in-house printers (Co. Formlabs, Form 2 and Form 3BL, USA), using Form 2 for the prototypes and Form 3BL for the production of the final model.

**Figure 3:**
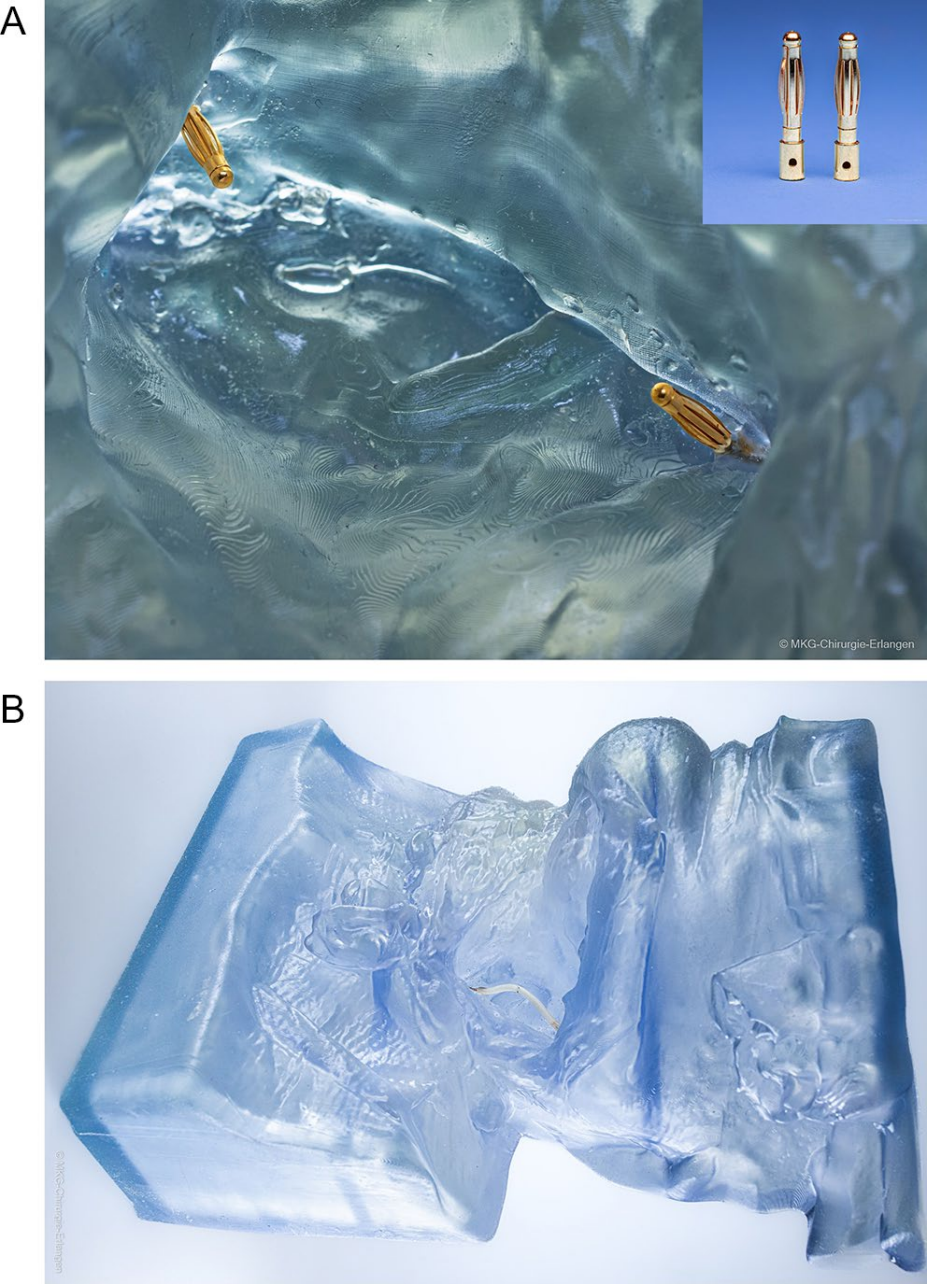
(**A**) Close-up view of the final RACE model. Close-up of the final RACE model showing the approximation of two artificial vessel stumps. Upper right corner: banana plugs used to anchor the vessels. (**B**) Front view of the final RACE model. Final RACE model (288 mm*207 mm*115 mm) with artificial micro-vessel after completion of microanastomosis.

Following printing, the parts were cleaned and finished by removing the support structures, rinsing with isopropyl alcohol and curing with 405 nm light according to the manufacturer’s instructions. After image magnification and identification of the connecting vessel to be simulated, two commercially available banana plugs (Co. Vuniversum, Goldkontaktstecker 2 mm, Germany) were anchored at the superior thyroid artery site for later placement of the training vessel by pre-drilling, wetting with resin (Co. Formlabs, Flexible Resin 80A or Elastic Resin 50A, USA) and manual curing (Fig. 3A). Curing can be performed using dental curing lights or commercially available laser pointers. Banana plugs were anchored 2 cm lateral to the original vessel position of the superior thyroid artery to ensure subsequent suturing in the immediate area of a likely connecting vessel for microsurgical anastomosis in head and neck surgery. Particular attention has been paid to the correct angulation of the plugs to ensure the correct angle of connection of the training vessel. Artificial microvessels of various calibres and types (e.g. Co. Limbs & Things, Bristol, UK /Co. WetLab Incorporated, Otsu-city, Japan and others) could then be attached to the model by clamping onto the anchored banana plugs. All methods were carried out in accordance with relevant guidelines and regulations. All experimental protocols including the clinical patient scan were approved by the Ethics Committee of the Friedrich-Alexander University Erlangen-Nuremberg which stated that no separate ethics application was necessary. Informed consent was obtained from all subjects.

Evaluation showed that the finished model (Fig. 3B) met the requirements described above for a realistic simulation of complex spatial-anatomical conditions, realistic haptic material properties, and economical and future-oriented development and implementation.

## Results

### RACE workflow step-by-step

As a result of the current work, we present the RACE workflow based on the microsurgical RACE model as a workflow for the development of general medical training models:

1. **R**egistration of the intraoperative anatomical surface structure using a suitable 3D scanner (e.g. Co. Artec, Eva, Germany).
2. **A**djustment and modelling of the obtained 3D data set using a 3D editing tool (e.g. Co. Autodesk, Meshmixer, USA).
3. **C**reation of the model in a suitable 3D printing process (e.g. stereolithography or polyjet technology).
4. **E**xtension with support structure to simulate the surgical procedure (e.g. support for microsurgical training vessels).

The workflow serves as a sequence of steps for the production of a simulation model. The first step is to acquire a 3D image of the surgical site or anatomical region of interest. Optical 3D scanners are recommended for this purpose due to their clinical practicality and ability to be used without exposure to ionising radiation. To generate a 3D model from the scan file, it must then be processed using an appropriate 3D modelling programme. Suitable tools for creating a printable volumetric body from a surface captured as an STL file include extrusion and offset operations. Once created, sidewalls and partitions are added to the volumetric body using simple combination or boolean union tools, depending on the printer's requirements. The model is then built using advanced additive manufacturing techniques. Stereolithography 3D printing offers an efficient and practical in-house solution. Polyjet technology allows multiple materials to be combined in a single model. To simulate different surgical techniques and teach procedural learning, an appropriate exercise task can be integrated into the model. In case of a microsurgical training model, this can be achieved by providing fixtures for microsurgical vessels.

### Features of the head and neck RACE model

**Figure 4:**
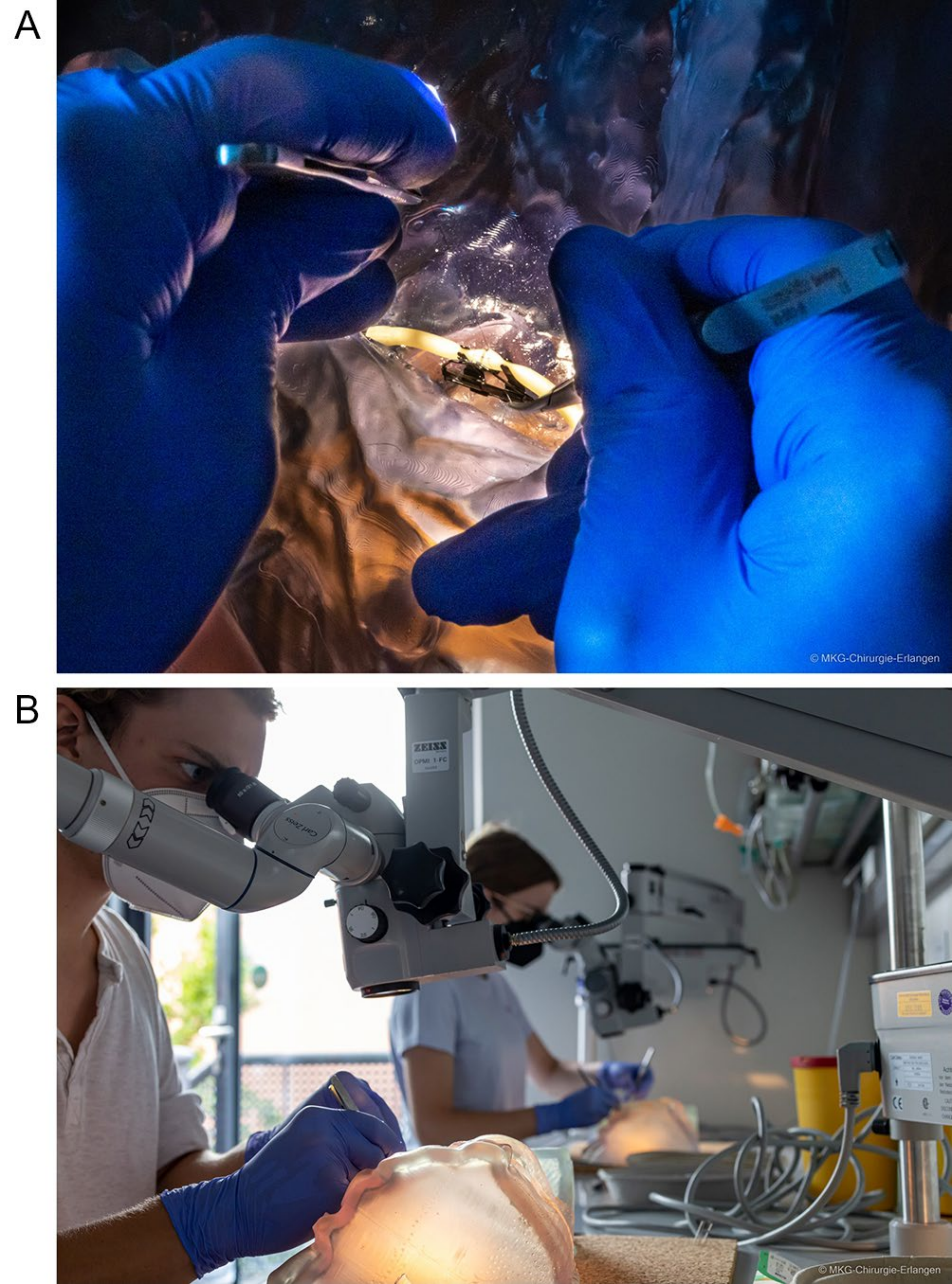
(**A**) Close-up view of instrument handling in restricted spatial conditions. The RACE model enables training of instrument, suture and needle handling in restricted spatial conditions. Unusual hand support techniques are acquired through training on the model. (**B**) Applying the model in student courses and resident training. The RACE model has been successfully established in student and resident training.

For the first time, the complex spatial-anatomical conditions of microanastomosis in the head and neck region can be adequately replicated in a training situation using the RACE model (Fig. 3B). The handling of instruments, sutures and needles as well as different support techniques can now be trained within limited spatial conditions. In addition, initial positioning of the microsurgical approximator and repositioning for suturing the back side can be trained under realistically complex and spatially constrained conditions (Fig. 4A and B). Experienced microsurgeons confirmed that the model had realistic spatial-anatomical features and effectively simulated complex training conditions.

To this date, we successfully established the RACE model in an undergraduate microsurgery course and resident training. Early evaluation results indicated an initial deterioration in instrument handling due to realistically complex conditions compared to the conventional teaching models (e.g. chicken thighs). This suggested a successful simulation of complex spatial-anatomical conditions. Further practice with the RACE model showed that the final training success is higher than that achieved with the conventional model in the same amount of time. The current RACE model with its final dimensions of 20.8 cm × 12.2 cm × 28.8 cm took 66 h to print at an average cost of 390 Euros. Once cured, it can be desinfected or discarded as normal. STL file of the final RACE model has been provided as open source for non-commercial use by the authors in the Supplementary Material section.

## Discussion

Current success rates for microsurgical tissue transfer in the head and neck region are estimated to exceed 90%^13,14^. However, loss of a microvascular graft is associated with significant functional and aesthetic limitations for the patient. Preoperative radiotherapy, intraoperative revision of the anastomosis and overall training and experience of the operating surgeon are the main factors contributing to the success of microsurgical tissue transfer^13,15,16^. In order to avoid graft loss and subsequent reoperation in patients who are often severely compromised, microsurgical teaching is a high priority in maxillofacial surgery as well as other microsurgical specialties. Most articles on microsurgical training are limited to the microanastomosis itself^3,4,7,8,10,17–19^—without considering the challenges associated with complex spatial-anatomical conditions which are particularly demanding in the head and neck region.

Traditionally, surgeons have been trained according to the Halstedian model of surgical education, in which the surgical craft is learned through hands-on practice on patients^20^. In comparison, training of pilots, soldiers and astronauts is much more advanced with the use of modern simulators to learn sophisticated skills^21–26^. Is contemporary surgical training still appropriate given the technical possibilities and the simultaneous responsibility towards the patient? Does surgery need to be learnt on the patient in order to be practised successfully and to meet expectations? According to de Montbrun and Macrae, the ethical imperative to provide the best possible care for patients at all times can be seen as a driving force behind the emerging trend shift towards simulation-based training in modern surgical education^27,28^.

There is ample evidence that bench training leads to skill acquisition. Jensen et al.^29^ showed that simulation-based training for excision of a skin lesion and small bowel anastomosis in a porcine model resulted in a significant improvement in performance with reduced time to completion, improved overall scoring and, for the anastomosis in particular, an increase in anastomotic leak pressure. The added value of high versus low-fidelity models is controversial^30–33^. For microvascular anastomosis training, Sidhu et al.^34^ demonstrated the superiority of high-fidelity models in a randomised controlled trial. In order to achieve optimal preparation for surgical work on patients, it can therefore be considered essential to create training conditions that are as close to reality as possible. Microsurgical animal models can be classified as high-fidelity models due to their ability to accurately simulate tissue properties rather than the spatial-anatomical environment^35^. Following the principle of the "3Rs", which is mandatory in animal experimentation: Replace, Reduce and Refine, we opted for a fully synthetic model without the use of animal cadavers. Regarding the choice of microvessels for the training model, Duraivel et al.^36^ recently presented an innovative silicon-based 3D printing process that can print fine blood vessels and other structures. However, the process requires highly specialised hardware. To make the model as widely applicable as possible, we decided to design it so that it could be printed on a standard 3D printer, adding commercially available and widely used simulation vessels. Experience from in-house student courses and resident training has shown that conventional or bench models are suitable for adequate teaching of microsurgical techniques and tissue handling. However, realistic spatial-anatomical conditions and their consequences in terms of lack of space, limited visibility and ergonomics are completely disregarded. We have developed, implemented and established the RACE model to teach microsurgical techniques under realistic spatial-anatomical conditions, thereby increasing surgeon confidence and performance under challenging intraoperative conditions. The 1:1 reproduction of a real microsurgical site allows suturing techniques to be taught under realistic and therefore didactically more valuable conditions compared to conventional models. Key challenges include difficult hand support in sloping areas, generally limited space and instrument handling in deep and partially submerged areas of the surgical site. The current RACE model for head and neck anastomosis simulates the neck site of a robust male. This could be followed by a slender female and a paediatric site model, including the simulation of different vessel connection sites.

The RACE workflow, developed on the basis of the RACE model, now enables the above aspects and the transfer of complex anatomical conditions of different patients into a teaching environment not only for microsurgical but also for general medical teaching. It is a departure from the Halstedian model of surgical teaching and addresses the anatomical variability of the human body and its challenges through realistic simulation. To date, patient specific anatomical variability has received initial attention in descriptive medical teaching^37^, less so in direct training and simulation of procedural skills. Wertz et al.^38^ introduced the element of “personalised medicine” to dental surgery education, where individual patients can be transformed into surgical training models by segmentation of the bony structure from preoperative CT scans. The RACE workflow now allows for additional soft tissue replication through direct surface scanning, providing an accurate simulation of the surgical site.

General medical training and surgical specialisation extend over a long period of time. Research has shown that older adults, even when given much more time to practice, do not acquire procedural skills at a level of performance comparable to that of younger adults^39^. The RACE workflow helps to integrate microsurgical, surgical and general medical teaching at an early stage and thus efficiently into the daily training of students and residents. Interestingly, although not reaching the performance levels of their younger counterparts^40,41^, older subjects can achieve similar effects through repetition of trials, in turn enabled by the reusable and sustainable nature of the model.

For the first time in microsurgery training, we have created an economical and practical method of combining surgical practice and education through the 1:1 replication of surgical sites, including precise soft tissue surface detail and opportunities for realistic hand support and suturing. This was realised by leveraging modern 3D scanning and printing technology. The RACE workflow, developed and established on the basis on our microsurgical RACE model, can serve as a template for other surgical skills and medical disciplines.

## Conclusion and outlook

The RACE model breaks with the Halstedian model of surgical education by realistically simulating the complex spatial-anatomical surgical environment in microsurgical training. The RACE workflow allows 1:1 transfer of surgical sites into the teaching environment and is applicable to various surgical specialties as well as general medical education. In addition to its sustainability, the RACE workflow allows for the transfer of variable surgical sites and different patients into the training situation, thus accommodating the anatomical diversity of our patient population. The model will be evaluated in future student and resident training using a competency-based assessment tool.

### Supplementary Information

Supplementary Information 1.

## Data Availability

All available data are presented in the manuscript.

## Abbreviations

RACE: Realistic Anatomical Conditions Experience
STL: Stereolithography
3D: Three-dimensional
